# Glucose Depletion in the Airway Surface Liquid Is Essential for Sterility of the Airways

**DOI:** 10.1371/journal.pone.0016166

**Published:** 2011-01-20

**Authors:** Alejandro A. Pezzulo, Jeydith Gutiérrez, Kelly S. Duschner, Kelly S. McConnell, Peter J. Taft, Sarah E. Ernst, Timothy L. Yahr, Kamal Rahmouni, Julia Klesney-Tait, David A. Stoltz, Joseph Zabner

**Affiliations:** 1 Department of Internal Medicine, University of Iowa, Iowa City, Iowa, United States of America; 2 Department of Microbiology, University of Iowa, Iowa City, Iowa, United States of America; University of Pittsburgh, United States of America

## Abstract

Diabetes mellitus predisposes the host to bacterial infections. Moreover, hyperglycemia has been shown to be an independent risk factor for respiratory infections. The luminal surface of airway epithelia is covered by a thin layer of airway surface liquid (ASL) and is normally sterile despite constant exposure to bacteria. The balance between bacterial growth and killing in the airway determines the outcome of exposure to inhaled or aspirated bacteria: infection or sterility. We hypothesized that restriction of carbon sources –including glucose– in the ASL is required for sterility of the lungs. We found that airway epithelia deplete glucose from the ASL via a novel mechanism involving polarized expression of GLUT-1 and GLUT-10, intracellular glucose phosphorylation, and low relative paracellular glucose permeability in well-differentiated cultures of human airway epithelia and in segments of airway epithelia excised from human tracheas. Moreover, we found that increased glucose concentration in the ASL augments growth of *P. aeruginosa in vitro* and in the lungs of hyperglycemic *ob/ob* and *db/db* mice *in vivo*. In contrast, hyperglycemia had no effect on intrapulmonary bacterial growth of a *P. aeruginosa* mutant that is unable to utilize glucose as a carbon source. Our data suggest that depletion of glucose in the airway epithelial surface is a novel mechanism for innate immunity. This mechanism is important for sterility of the airways and has implications in hyperglycemia and conditions that result in disruption of the epithelial barrier in the lung.

## Introduction

Hyperglycemia in both diabetic and non-diabetic patients is associated with an increased risk of complications and mortality from community acquired pneumonia [1]–[3]. Interestingly, in the absence of hyperglycemia, diabetes itself does not increase these risks [2]–[4]. A number of possible explanations for the association between hyperglycemia and pulmonary infection exist, but the mechanisms are still unknown.

The luminal surface of airway epithelia is covered by a thin layer of fluid, termed the airway surface liquid (ASL) [5], and is normally sterile despite frequent exposure to bacteria, fungi and viruses. Inhaled and aspirated bacteria [6] are cleared upon reaching this surface by mechanisms that include ASL antimicrobials, mucociliary clearance and other components of innate and adaptive immunity. In addition to these antimicrobial components, the ASL also contains factors that are required for bacterial growth, such as electrolytes, proteins, lipids, amino acids and oligo-/mono-saccharides, including glucose [7], [8]. It has been speculated that amino acid, peptide and sugar residue transporters contribute to lung host defense by clearing these compounds from the surface of the airways and alveoli and making them unavailable to bacteria [8], [9].

Recently, Baker *et al*. [10] showed that the concentration of glucose in the ASL is increased in hyperglycemic non-diabetics and diabetics, and in humans with cystic fibrosis (CF). In other studies, the same group demonstrated that the detection of glucose in bronchial aspirates of intubated patients correlated with the risk of MRSA infection [11] and that glucose in the nasal secretions of humans with CF-related diabetes facilitated growth of *Staphylococcus aureus* and *Pseudomonas aeruginosa* [12]. In this study, we hypothesized that restriction of carbon sources -specifically glucose- in the ASL is an important component of innate immunity. We report a mechanism responsible for a glucose concentration gradient generated by human airway epithelia resulting in low ASL glucose concentration, impaired bacterial growth, and airway sterility.

## Materials and Methods

For detailed materials and methods, see Supporting Methods in Text S1.

### Ethics Statement

Animal experiments were carried out in strict accordance with the recommendations in the Guide for the Care and Use of Laboratory Animals of the National Institutes of Health. All experiments were approved by the Animal Care and Use Committee of the University of Iowa (ACURF # 1004078).

### Primary Cultures of human airway epithelia

Primary human airway epithelia were grown at the air-liquid interface as previously described [13].

### Transmission Electron Microscopy

Primary cultures of human airway epithelia were fixed using a fluorocarbon-based protocol that allows preservation of the ASL and mucus layer.

### Collection of airway surface liquid (ASL)

ASL was collected from primary cultures of human airway epithelia and glucose concentration was determined using a glucose oxidase assay.

### Measurement of transepithelial electrical properties of intact human tracheal epithelia

Epithelia dissected from 3 human tracheas (obtained from the University of Iowa In Vitro Models and Cell Culture Core) were studied in Ussing chambers.

### Microarray data analysis

A MIAME-compliant microarray dataset previously deposited in GEO under accession #GSE20502 was used to determine the mRNA expression profile of GLUT and SGLT transporters in human airway epithelia *in vivo* and *in vitro*. For method details, see GEO accession.

### Immunocytochemistry

Primary cultures of HAE were immunostained and visualized with confocal microscopy as described in [14]. For primary and secondary antibodies, see Text S1. As controls, we studied epithelia without primary antibody and isotype-matched control antibody.

### Bidirectional fluxes and uptake of glucose

Bidirectional fluxes and uptake of radiolabeled L-glucose and 2-deoxyglucose were done using glucose-free DMEM supplemented with 5 mM of the corresponding non-radiolabeled and 0.5 µCi of radiolabeled glucose analog.

### Bacterial strains

The PAO1 strain of *P. aeruginosa* was used where indicated. The *edd* ^−^ mutant was obtained from the UW *P. aeruginosa* Mutant Library [15]. Adequate transposon insertion was confirmed by PCR as indicated by providers of the strain.

### *P. aeruginosa* growth *in vitro*

PAO1 was grown overnight in LB medium at 37°C. Bacteria were centrifuged at 4°C for 15 min. at 3200× g. and resuspended in 50 mL M9 medium twice followed by dilution in M9 or ASL pooled from 6 different donors, to an OD 0.05 at 600 nm. Glucose was added as indicated. Samples were kept in a shaking incubator at 300 rpm and 37°C, and OD 600 nm was determined at times shown.

### *P. aeruginosa* growth on cultures of human airway epithelia

The apical surface of cultures of human airway epithelia was inoculated with PAO1 in a volume of 0.1 µL and bacteria were quantified after 24 hours.

### *P. aeruginosa* pulmonary challenge in mice

Sex-matched 22 to 24 week-old *ob/ob* and *db/db* mice and their littermate controls were used. Intranasal inoculation with the indicated bacterial strain was followed by quantification of bacteria in lung homogenate after 6 hours.

### Statistical analysis

Statistical analyses were performed in Graphpad Prism 5 for MacOSX. Unpaired T-test was performed on every analysis except in Figure 4, C and D, where a Z-test for proportions was used and Figure 5, A and B, where Mann-Whitney U test for nonparametric data was performed. A p-value <0.05 was considered statistically significant [16].

## Results

### Airway epithelia generate a transepithelial glucose gradient

**Figure 1:**
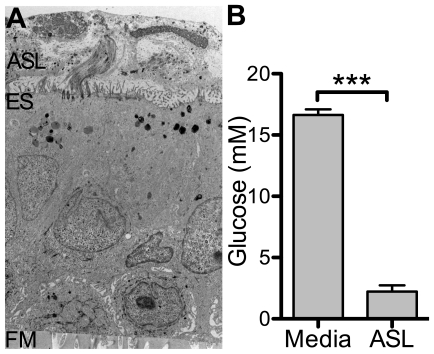
Human airway epithelia maintain a transepithelial glucose gradient. (**A**) Transmission electron microscopy of perfluorocarbon-fixed cultures of human airway epithelia. Figure indicates airway surface liquid (ASL), epithelial surface (ES) and filter membrane support (FM). (**B**) Glucose concentration in ASL collected from cultures of human airway epithelia was compared to glucose concentration in basolateral nutrient media. Data shown as mean ± s.e.m. n = 6 samples per group. (***: p<0.0001).

Studies of exhaled breath condensate have shown that the glucose concentration in ASL is approximately one tenth that in serum [10]. We hypothesized that the airway epithelium can maintain a transepithelial glucose gradient. We collected ASL from well-differentiated human airway epithelia cultures grown at the air-liquid interface (Figure 1, A), and compared the glucose concentration to that in the basolateral nutrient media. While the basolateral glucose concentration was 16.6±0.4 mM, the concentration in the ASL was 2.2±0.5 mM (Figure 1, B).

### Na^+^/glucose co-transporter activity is absent from airway epithelia

**Figure 2:**
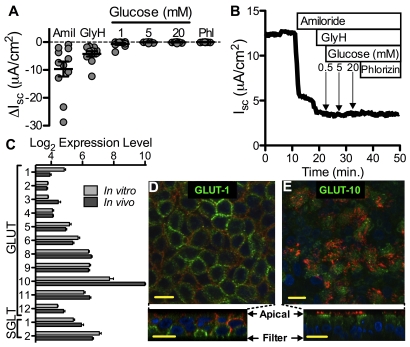
Human airway epithelia express GLUT-1 and GLUT-10. (**A**) Short-circuit current (I_sc_) in human bronchial epithelia was studied in Ussing chambers. To maximize detection of SGLT-mediated sodium absorption, amiloride and GlyH were added to the apical buffer to block ENaC and CFTR-mediated absorption of sodium and secretion of chloride, respectively, followed by glucose in increasing concentrations and phlorizin. Each circle corresponds to one sample (11 total) obtained from 3 donors. Mean ± s.e.m are shown (**B**) Representative I_sc_ tracing from intact human bronchial epithelia. (**C**) Microarray analysis of mRNA from *in vivo* and *in vitro* human airway tissue. Normalized expression levels of GLUT and SGLT gene family members are shown. n = 16 samples per group. (**D and E**) Cultures of human airway epithelia were immunostained using a GLUT-1 (D green) or GLUT-10 (E, green) antibody. XY (top image) and XZ (bottom image) projections are shown. DAPI (nuclei) is shown in blue, E-cadherin (intercellular junction) is shown in D (red), and acetylated alpha tubulin (cilia) is shown in E (red). Representative images from 3 experiments are shown. Isotype-matched antibody and no-primary controls did not show fluorescence after staining. Scale bars, 10 µm.

Na^+^-glucose co-transporters (SGLT-1 and SGLT-2) transport glucose against a concentration gradient in intestinal, renal, and lung alveolar epithelia [17], [18]. Transport of glucose follows the Na^+^ gradient generated by the Na^+^/K^+^ ATPase pump. This mechanism is capable of generating a current that can be measured as a short circuit current (I_sc_) in Ussing chamber studies [19]. We hypothesized that glucose depletion from the ASL occurs through SGLT-mediated Na^+^-glucose co-transport. Airway epithelia dissected from human tracheas were mounted in Ussing chambers for I_sc_ measurements. Apical glucose concentration was sequentially increased (Figure 2, A–B). Apical glucose did not generate a change in I_sc_, in contrast to tissues that express Na^+^-glucose co-transporters (i.e. jejunum of mice and alveolar epithelial cells of rats) where, as previously shown, addition of glucose to the apical surface results in a change in I_sc_ [20], [21]. Moreover, addition of phlorizin, an SGLT blocker, did not result in a decrease in I_sc_, further confirming absence of Na^+^-glucose co-transport activity. Complementary experiments were performed using well-differentiated cultures of human airway epithelia (Figure S1) and results are consistent with data from intact airway epithelia. These data suggest that electrogenic Na^+^-glucose co-transport is not the mechanism that generates the transepithelial glucose gradient in human airway epithelia.

### Polarized expression of glucose transporters in human airway epithelia

To identify glucose transporters in human airway epithelia, we first performed a screening analysis of GLUT (glucose facilitated diffusion proteins) and SGLT mRNA expression. We analyzed a dataset (GEO Accession #GSE20502) derived from microarray hybridizations with human *in vivo* and *in vitro* airway tissue (Figure 2, C). The analysis revealed a high correlation between expression levels of glucose transporters *in vitro* and *in vivo* (R^2^ = 0.81). We noticed high-level expression of GLUT-10 (Entrez GeneID: 81031), which is encoded by a 5-exon gene, located on chromosome 20. GLUT-10 mRNA has previously been detected in lung tissue and is also expressed in the heart, brain, skeletal muscle and placenta [22], [23].

We then investigated the expression and localization of glucose transporters in human airway epithelia by immunocytochemistry. We found no expression of GLUT-2, GLUT-3, GLUT-4, GLUT-8, SGLT-1 or SGLT-2. We detected GLUT-1 (Entrez GeneID: 6513) in the basolateral membrane (Figure 2, D), and GLUT-10 localized to the apical membrane of human airway epithelia (Figure 2, E). These data suggest that expression of different facilitated diffusion glucose transporters in the apical and basolateral membranes of human airway epithelia may play a role in generation of the transepithelial glucose concentration gradient.

### Bidirectional fluxes of glucose in human airway epithelia

The existence of a transepithelial glucose concentration gradient in the absence of active sodium-coupled absorption suggests that either 1) basolateral glucose supplied to the epithelial cells is unable to reach the ASL through the paracellular or the transcellular pathway, unlikely given that even in “tight epithelia”, paracellular permeability to small molecules is not 0 (the paracellular pathway is dynamic) or 2) At equilibrium, the transepithelial flux of glucose into ASL is balanced by an absorptive process.

**Figure 3:**
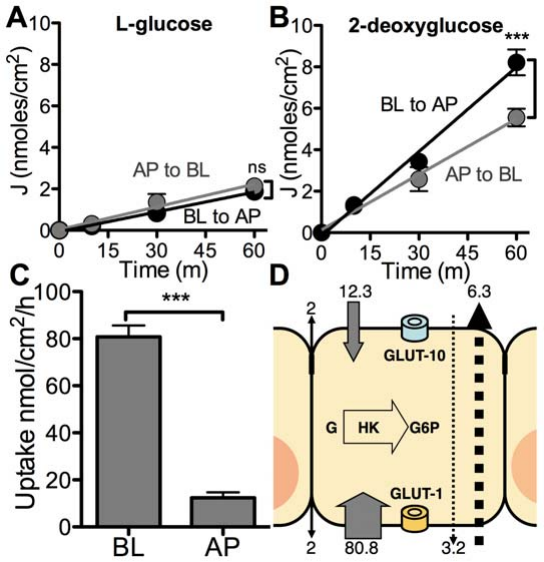
Airway epithelia can deplete airway surface liquid glucose. (**A and B**) Basolateral to apical (BL to AP) and apical to basolateral (AP to BL) fluxes of L-[1-^14^C]glucose (**A**) or 2-deoxy-d-[1-^14^C]glucose (**B**) were measured in cultures of human airway epithelia over 1 hour. Data shown as mean ± s.e.m. n = 6 samples per group. (***: p<0.0001, ns: p ≥ 0.05). (**C**) Basolateral (BL) and apical (AP) uptake of 2-deoxy-d-[1-^14^C]glucose (2-DOG) in cultures of human airway epithelia were measured over 1 hour. Data shown as mean ± s.e.m. n = 6 samples per group. (***: p<0.0001). (**D**) Data from Figures 3 A, B and C are integrated into a model of glucose transport in human airway epithelia. Data in nmol/h. Black solid arrow represents paracellular diffusion of glucose. Gray solid arrows represent 2-DOG uptake capacity across the apical (top) and basolateral (bottom) membranes. Dotted arrows represent transcellular bidirectional fluxes of 2-DOG (adjusted by subtracting corresponding L-glucose fluxes). GLUT-10 is present in the apical membrane and GLUT-1 is present in the basolateral membrane. Intracellular glucose (G) is phosphorylated by hexokinase (HK) for subsequent glycolysis. G6P =  glucose-6-phosphate.

We first measured the bidirectional flux of ^14^C-L-glucose in human airway epithelia. L-glucose is a stereoisomer of D-glucose that allows determination of paracellular transport of glucose because it is not a substrate for glucose transporter proteins. The ^14^C-L-glucose transport rate (Figure 3, A) was similar in both directions (basolateral to apical  = 1.90±0.09 nmoles/cm^2^/h, apical to basolateral  = 2.19±0.24 nmoles/cm^2^/h, p = 0.24) indicating paracellular permeability to glucose but no net directional transport. To calculate transcellular glucose transport, we repeated the bidirectional flux assays using ^14^C-2-deoxyglucose (^14^C-2-DOG), a glucose analog that is a substrate for glucose transporters and can also be phosphorylated by the intracellular hexokinase activity of the airway epithelial cells [24]. Once phosphorylated, glucose or 2-DOG cannot be transported across the cell membrane. We found a basolateral to apical transport rate (8.15±0.52 nmoles/cm^2^/h) and an apical to basolateral transport rate (5.35±0.38 nmoles/cm^2^/h) (Figure 3, B) that were both higher than that observed for paracellular transport. These results indicate that glucose supplied to airway epithelia can reach the ASL through both the paracellular and transcellular pathway. We next hypothesized that the apical glucose uptake capacity would be equal or greater than the net basolateral to apical transepithelial flux of glucose.

### Airway epithelia absorb glucose from the apical and basolateral compartments

We conducted radiolabeled tracer uptake experiments in which we measured the amount of glucose that entered the intracellular compartment after addition to the apical or basolateral side of well-differentiated cultures of human airway epithelia. Uptake of ^14^C-2-DOG per sample was 12.3±2.4 nmoles/cm^2^/h after apical addition and 80.8±4.8 nmoles/cm^2^/h after basolateral addition (Figure 3, C). These data show that in human airway epithelia, both the apical and the basolateral membrane are permeable to glucose and basolateral uptake occurs at a higher rate than apical uptake of glucose. Therefore, although glucose can reach the ASL through both the paracellular and transcellular pathways at a specific rate, the epithelial cells have the capacity to absorb it through the apical membrane at an equal or higher rate (Figure 3, D). This would allow the presence of a transepithelial glucose gradient at homeostasis and in the absence of Na^+^-glucose co-transport activity, as detailed in the discussion section. As a comparison, we investigated glucose fluxes in *ex vivo* tracheal epithelia from *Sus scrofa* (domestic pig) in Figure S2. The data indicate that in *ex vivo* tracheal epithelia, glucose can reach the ASL through the paracellular but not the transcellular pathway and uptake occurs at both the apical and basolateral membranes. These results are concordant with our *in vitro* data. We next studied the physiological significance of this transepithelial glucose gradient.

### Low glucose concentration impairs bacterial growth in ASL

**Figure 4:**
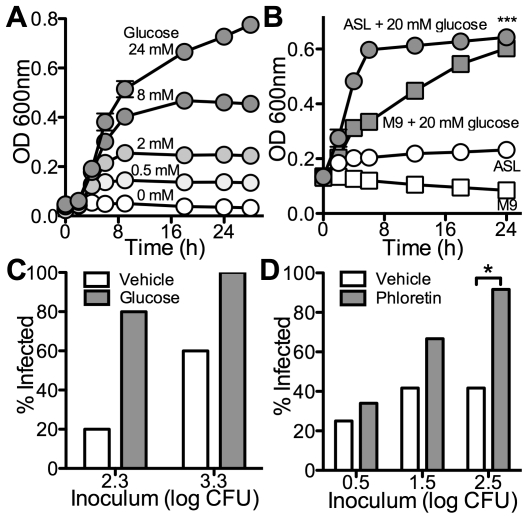
Low glucose concentration impairs growth of *P. aeruginosa in vitro*. (**A**) Glucose concentration-dependent growth of *P. aeruginosa*. M9 medium with glucose concentrations as shown (0, 0.5, 2, 8 and 24 mM) was inoculated with *P. aeruginosa* (PAO1) and OD600 was followed. Data shown as mean ± s.e.m. n = 3. (**B**) Growth of *P. aeruginosa in* ASL is nutrient-limited. ASL and M9 medium with and without added glucose (20 mM) as shown were inoculated with PAO1. OD600 was followed over time. Data shown as mean ± s.e.m. n = 3. (***: p<0.0001 for ASL vs. ASL + glucose). (**C**) Growth of *P. aeruginosa* in human airway epithelia is limited by ASL glucose concentration. Cultures of human airway epithelia were inoculated apically with 2.3 or 3.3 log CFU of PAO1 in the absence (white bars) or presence (gray bars) of added glucose (20 mM). After 24 h, apical bacteria were quantified. Percentage of samples in which growth of bacteria occurred are shown. n = 5. (**D**) Facilitated diffusion transport in airway epithelia *in vitro* impairs growth of *P. aeruginosa.* Cultures of human airway epithelia were inoculated apically with 0.5, 1.5 or 2.5 log CFU of PAO1 after incubation with apical vehicle (white bars) or phloretin (gray bars). After 24 h, apical bacteria were quantified. Percentage of samples in which growth of bacteria occurred are shown. n = 12. (*: p<0.05 for vehicle vs. phloretin).

Airway surface liquid contains multiple antimicrobials known to play a role in innate immunity [7], [25]. We hypothesized that the low glucose concentration in ASL may result in an impaired bacterial growth rate, favoring antimicrobial activity over bacterial growth. We chose *P. aeruginosa* (strain PAO1) as a pathogen because it can use multiple carbon sources for growth, including glucose [26]. The concentration of glucose in minimal growth media (M9) directly determined the growth rate of PAO1 in a dose-dependent manner (Figure , A4). When we inoculated ASL *in vitro* (Figure 4, B), PAO1 grew only in the presence of 20 mM glucose (mean OD at 24 h for ASL = 0.231±0.008 vs. mean OD at 24 h for ASL + glucose = 0.643±0.012. p<0.0001). The growth rate observed without added glucose is in the range seen in minimal growth media with 0.5 to 2 mM glucose. These data suggest that ASL does not contain a sufficient concentration of carbon sources to support growth of *P. aeruginosa*.

### Low glucose concentration impairs bacterial growth on the airway surface *in situ*

Airway surface liquid kills bacteria using salt-sensitive peptides such as lysozyme and defensins [7], [25] that are efficient only against small inoculum sizes [25], [27]. An increased bacterial growth rate in the presence of excess nutrient sources could overwhelm this system by allowing the bacteria to reach the threshold at which innate immune killing becomes ineffective. We investigated the effect of glucose on the ability of PAO1 to grow on the surface of human airway epithelia. We predicted that increasing the glucose concentration in ASL would impair the bactericidal activity against PAO1. We inoculated the apical surface of human airway epithelia cultures from 5 different donors with a PAO1 inoculum of 2.3 or 3.3 log CFU per group. As previously shown [27], human airway epithelia can effectively kill 2.3 log CFU of *P. aeruginosa* but not 3.3 log CFU. Importantly, when the inoculum and glucose were simultaneously applied to the ASL, airway epithelia failed to kill even 2.3 log CFU of PAO1 (Figure 4, C). We then predicted that lack of apical glucose transport would result in increased bacterial growth in ASL. While human airway epithelia were able to effectively kill an inoculum of up to 2.5 log CFU, preincubation with 5 mM apical glucose and phloretin –an inhibitor of GLUT transporters– resulted in failure to kill inoculums of 1.5 and 2.5 log CFU (Figure 4, D). These data are consistent with our *in vitro* results and suggest that low glucose concentration in ASL is important in preventing bacterial growth on the airway surface.

### Hyperglycemia promotes bacterial growth in the lungs of mice

To test the significance of low glucose concentrations in the ASL *in vivo*, we studied PAO1 growth in murine lungs. Collecting undiluted ASL from mouse airways is technically challenging. It has been shown [28] that attempts to collect ASL using filter paper techniques result in submucosal liquid collection with little ASL recovery. Therefore, to investigate the effect of hyperglycemia on ASL innate immunity, we used three complementary approaches that include two models of hyperglycemic mice and a mutant strain of *P. aeruginosa* that cannot metabolize glucose.

**Figure 5:**
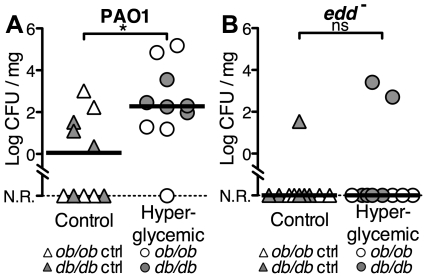
Hyperglycemia promotes growth of *P. aeruginosa* in the lungs of mice. (**A and B**) An intranasal inoculum (7.69 log CFU) of PAO1 (**A**) or *edd* ^−^ (**B**) was given to *ob/ob* and *db/db* mice and their respective littermate controls (*ob/ob* ctrl and *db/db* ctrl). After 6 hours, lungs were homogenized for bacterial counts. n = 5 mice per group. (*: p<0.05, ns: p ≥ 0.05).

First, we investigated the intrapulmonary growth of PAO1 (Figure 5, A) in *ob/ob* mice, which are genetically leptin deficient and become hyperglycemic and obese [29]–[33]. An intranasal inoculum of 7.69 log CFU of PAO1 was given to *ob/ob* mice (serum glucose = 20.7±4.7 mM) and their littermate controls (serum glucose = 5.7±1.2 mM). Bacterial counts in lung homogenates 6 hours after inoculation were higher in hyperglycemic *ob/ob* mice than in controls (median log CFU/mg of lung tissue  = 1.28 for *ob/ob* vs. 0 for control mice).

Second, since the observed differences in the previous experiment could be a direct consequence of leptin deficiency, we repeated the experiment (Figure 5, A) using *db/db* mice [30], [34] and their littermate controls. These mice are leptin-receptor deficient and also become hyperglycemic and obese (serum glucose = 22.6±5.3 mM). Bacterial counts in lung homogenates 6 hours after inoculation were higher in hyperglycemic *db/db* mice than in their littermate controls (median log CFU/mg of lung tissue = 2.3 for *db/db* vs. 0.35 for control mice). The data from *ob/ob* and *db/db* mice show that the bacterial counts are significantly different between hyperglycemic and control mice (median log CFU/mg of lung tissue = 2.27, IQR 1.26 – 4.3 for hyperglycemic mice vs. median = 0.05, IQR 0 – 1.82 for control mice, p = 0.0176). The bacterial counts are not significantly different when the *db/db* genotype is compared to the *ob/ob* genotype (p = 0.848) excluding leptin or leptin-receptor deficiency as the cause of the difference in bacterial counts between control and hyperglycemic mice.

Finally, we hypothesized that the higher susceptibility of hyperglycemic mice to infection is caused by elevated concentrations of glucose on the surface of the airway resulting in increased availability of glucose as a nutrient source for bacteria. We predicted that we would see no difference in bacterial growth if we used a mutant of PAO1 incapable of metabolizing glucose. Some artificially generated mutants [15] or clinical strains [12] of *P. aeruginosa* show variability in their capacity to preferentially use glucose as a carbon source, allowing to specifically assess the role of glucose in bacterial growth *in vivo*. We repeated the experiment using a transposon insertion mutant of PAO1 [15] with a defective *edd* gene, which codes for phosphogluconate dehydratase, a protein required for the catabolism of glucose and gluconate to glyceraldehyde-3-phosphate and pyruvate via the Entner-Doudoroff pathway [26], [35], [36]. The *edd* ^−^ mutant was phenotypically similar to PAO1 in assays of swimming motility, twitching motility, protease activity and Type III secretion system protein (PcrV and ExoS) expression (Figure S3). In addition, lethality in a *Drosophila melanogaster* infection model was also similar following *edd* ^−^ and PAO1 infection (Figure S4). When *edd* ^−^ was used (Figure 5, B) to inoculate the airways of *ob/ob* mice and *db/db* mice and their respective littermate controls, neither showed increased bacterial growth when compared to their normoglycemic controls (median log CFU/mg of lung tissue = 0, IQR 0 – 0 for control mice vs. median = 0, IQR 0 – 0.67 for hyperglycemic mice, p = 0.5036). In contrast, when the *edd* ^−^ mutant strain was injected into the peritoneal cavity (a nutrient-rich environment) of mice, the LD50 was similar to that of PAO1 (Figure S5). These data show that increased susceptibility to PAO1 infection in hyperglycemic mice is due to the elevated glucose concentration in ASL, which can be used by the bacteria as a nutrient source, and confirms that low glucose concentration in the ASL is required for the antimicrobial activity of airway epithelia *in vivo*.

## Discussion

In this study, we describe a novel mechanism that allows human airway epithelia to generate a transepithelial glucose concentration gradient. Moreover, this gradient results in an ASL that contains a low glucose concentration. Our studies suggest that this low concentration of glucose plays an important role in limiting the growth of bacteria and maintaining the sterility on the surface of human airway epithelia and leads us to speculate that the concentration of other carbon sources in ASL may also be low.

We propose a novel mechanism in human airway epithelia that generates a transepithelial glucose concentration gradient by expressing 2 distinct facilitated diffusion transporters in polarized localizations. This is in contrast to mechanisms in epithelia of the human ileum or alveolar epithelia in the distal lung, where Na^+^-glucose cotransport plays a central role in transepithelial glucose transport. GLUT-1, which we observed in the basolateral membrane, is the most ubiquitously expressed facilitated diffusion glucose transporter in humans; it has a K_m_ between 3 and 7 mM and has a proposed function of cellular insulin-independent basal glucose uptake [18], [37]. GLUT-10, which we observed in the apical membrane of human airway epithelia, has previously been examined because of a potential link to Diabetes Mellitus Type 2, but studies failed to prove an association [38]–[40]. Interestingly, GLUT-10 has a very low K_m_ of approximately 0.3 mM, the lowest in the group of facilitated diffusion glucose transporters [18], [22]. This K_m_ is very close to the glucose concentration in both human ASL [10] and rat lung cells [41] (0.5 mM).

Glucose is exclusively supplied to the airways from circulating blood, reaching the basolateral side of epithelial cells, where uptake of glucose can occur. Glucose can reach the ASL through both the paracellular and transcellular pathways at a specific rate, but the epithelial cells have the capacity to absorb it through the apical membrane at an equal or higher rate (Figure 3D).

**Figure 6:**
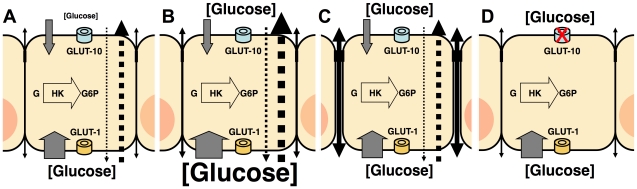
Alterations in transepithelial glucose transport. Diagram of transepithelial glucose transport under normal conditions (**A**), hyperglycemia (**B**), increased tight junction permeability (**C**) or impaired apical glucose transporter function (**D**). Gray solid arrows represent glucose uptake capacity across the apical (top) and basolateral (bottom) membranes. Dotted arrows represent transcellular bidirectional fluxes of glucose. Arrow width and font size are modified in each figure according to the primary alteration in glucose transport rate and resulting basolateral and apical concentration, respectively. GLUT-10 is present in the apical membrane and GLUT-1 is present in the basolateral membrane. Intracellular glucose (G) is phosphorylated by hexokinase (HK) for subsequent glycolysis. G6P =  glucose-6-phosphate.

What is the driving force that allows epithelial absorption of glucose from both the apical and basolateral compartments? Our data supports interesting conclusions. Intracellular glucose, under normal conditions, is constantly phosphorylated by hexokinase in an ATP-dependent reaction, creating flux into a chemical “fourth compartment”. This would maintain an intracellular non-phosphorylated glucose concentration that is lower than that of blood, and a glucose concentration gradient that can be the driving force for basolateral uptake. Any glucose reaching the ASL would therefore be absorbed at a rate that would keep it at concentration equilibrium with intracellular non-phosphorylated glucose (Figure 6, A). Data supporting this idea come from studies performed in isolated lung cells grown in suspension, in which the intracellular concentration of glucose was found to be <0.5 mM when the extracellular glucose was 5 mM or higher [41].

Disruption of this homeostatic mechanism could occur at different levels. An increase in blood glucose concentration, such as in diabetes mellitus, could result in an increased rate of glucose flux into ASL with correspondingly increased glucose concentration (Figure 6, B). This could explain the increased rate of ventilator-associated MRSA pneumonia [11] in ICU patients with hyperglycemia and the poor outcomes in hyperglycemic patients hospitalized for community acquired pneumonia [3]. This mechanism could also be disrupted by fluctuations of tight junction permeability (Figure 6, C), such as those induced by hypersensitivity reactions, exposure to toxic particles, or other inflammatory stimuli, including viral infections such as influenza or the common cold, which have been shown to increase the risk of bacterial pneumonia and correlate with detection of nasal glucose [1], [10], [42]–[45]. Also, even though environmental exposures to glucose are uncommon (confectioners lung), the presence of sugars in formulations that are nebulized into the airway could affect the homeostatic mechanism we have described. Finally, disruption of apical transport of glucose –as shown in our *in vitro* data– could impair generation of the glucose concentration gradient (Figure 6, D). It has been shown that mutations in the gene encoding GLUT-10 are responsible for Arterial Tortuosity Syndrome [46], [47], a rare autosomal recessive disorder, with a phenotype characterized by arterial elongation, tortuosity and aneurysms, and a high mortality rate (40% before the age of 5 years). The cause of early deaths has not been studied extensively, but a lethal case of spontaneous bilateral *Staphylococcus aureus* bronchopneumonia in a 4 year-old child has been reported [48]. The question of whether this infection was a consequence of impaired glucose transport in the airways remains to be answered.

Bacteria constantly challenge the airways, and whether they are eliminated, resulting in a sterile lung, or proliferate to cause infection or colonization depends on host factors and the bacteria. In the airways, multiple innate immune mechanisms have evolved to rapidly kill, expel, or engulf bacteria. These diverse mechanisms act within seconds (antimicrobials, cough) or minutes (mucociliary clearance and phagocytes) and are most effective with small inocula of bacteria. Abnormally high glucose concentration in the ASL could both directly promote bacterial growth and impair antimicrobials [49]. When bacteria are not eliminated, growth can be exponential and virulence factors of pathogenic and non-pathogenic bacteria can play a role. Thus, in the airways, the time-dependent balance between bacterial killing and growth determine the outcome. Our data suggest that carbon source deprivation in airway surface liquid helps tip the balance towards airway sterility.

## Supporting Information

Figure S1 Na^+^-glucose cotransport activity is absent from well-differentiated cultures of human airway epithelia. Short-circuit current in cultures of human airway epithelia was studied in Ussing chambers. Amiloride and bumetanide were added to Ussing chamber solution, followed by glucose in the apical or basolateral solution in increasing concentrations. Representative tracings of 3 replicates are shown.
(TIFF)

Figure S2 Basolateral to apical (BL to AP) and apical to basolateral (AP to BL) fluxes of L-[1-^14^C]glucose (**A**) or 2-deoxy-d-[1-^14^C]glucose (**B**) were measured in intact pig tracheal epithelia over 1 hour. Data shown as mean ± s.e.m. n  =  9 samples per group. (***: p<0.0001, ns: p ≥ 0.05). (**C**) Basolateral (BL) and apical (AP) uptake of 2-deoxy-d-[1-^14^C]glucose (2-DOG) in intact pig tracheal epithelia were measured over 1 hour. Data shown as mean ± s.e.m. n  =  9 samples per group. (***: p<0.0001). (**D**) Correlation of transepithelial electrical conductance at baseline vs. transepithelial conductance of glucose in intact pig tracheal epithelia. In the intact tracheal epithelia of pigs, bidirectional fluxes of L-glucose were higher than in cultured human epithelia and fluxes of 2-DOG occurred at similar rates in the presence of uptake from both the apical and basolateral membranes. These data indicate that glucose supplied to intact airway epithelia can reach the ASL through the paracellular but not the transcellular pathway. Moreover, the result suggests that intracellular phosphorylation of glucose resulting in a transepithelial concentration gradient occurs at a higher rate *in vivo* compared to cultured cells.
(TIFF)

Figure S3 Swimming motility, twitching motility, protease activity and Type III secretion system protein expression are preserved in *edd* ^−^. (**A**) A swimming motility assay comparing PAO1 and *edd* ^−^ was performed as described in [50]. Representative sample of n  =  3 is shown. (**B**) A twitching motility assay comparing PAO1 and *edd* ^−^ was performed as described in [51], duplicate samples are shown. (**C**) Protease activity was characterized as described in [52], triplicate samples shown. (**D**) Expression of PcrV and ExoS, which are Type III secretion system proteins, was determined by using the method described in [53].
(TIFF)

Figure S4 *edd* ^−^ lethality is preserved in a *D. melanogaster* infection model. Survival of *D. melanogaster* was monitored for 72 h after inoculation of the abdominal cavity with PAO1 or *edd* ^−^ as described in [54] (n = 20).
(TIFF)

Figure S5 *edd* ^−^ lethality is preserved in a murine peritoneal infection model. All experiments were reviewed and approved by the Animal Care and Use Committee of the University of Iowa. Male 6 to 8 week old C57B/J6 mice obtained from Harlan Industries, Inc (Indianapolis, IN, USA) were used and were allowed access to food and water ad libitum. Mice were anesthetized with isoflurane and injected intraperitoneally with 3×10^7^ CFU of PAO1 or *edd* ^−^ in a volume of 100 μL H_2_O. *exsA* ^−^ strain of *P aeruginosa* was used as a negative control. Mice were carefully monitored and euthanized when end-point conditions were met (moribund, distressed, and unable to eat or drink).
(TIFF)

Text S1 Supporting Information Methods.
(DOC)
