# Supplementary figures and images for: Glucose Depletion in the Airway Surface Liquid Is Essential for Sterility of the Airways
Source: PLoS One. 2011 Jan 20;6(1):e16166. doi: 10.1371/journal.pone.0016166 (PMC3029092; doi:10.1371/journal.pone.0016166)

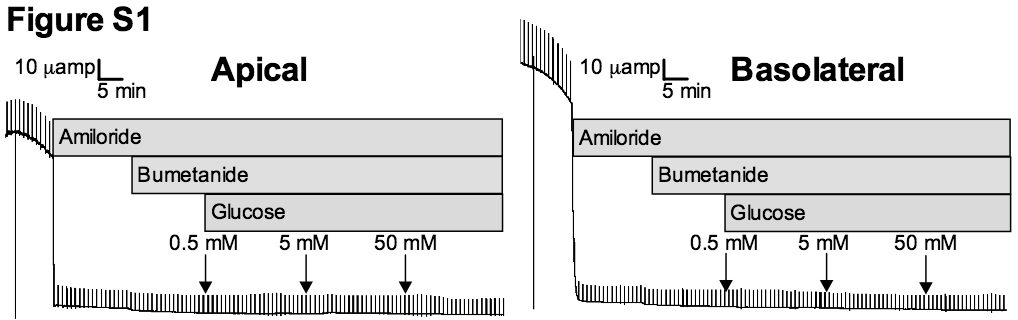

Supplement: Figure S1 — Na+-glucose cotransport activity is absent from well-differentiated cultures of human airway epithelia. Short-circuit current in cultures of human airway epithelia was studied in Ussing chambers. Amiloride and bumetanide were added to Ussing chamber solution, followed by glucose in the apical or basolateral solution in increasing concentrations. Representative tracings of 3 replicates are shown. (TIFF) [file pone.0016166.s001.tif]

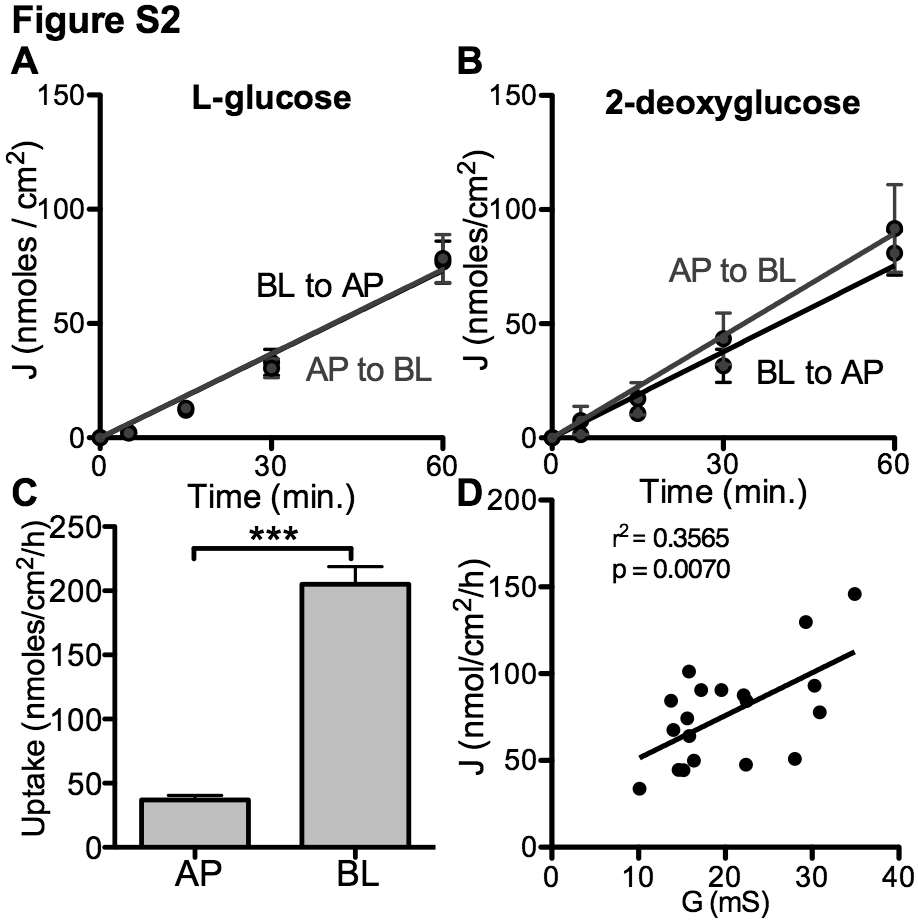

Supplement: Figure S2 — Basolateral to apical (BL to AP) and apical to basolateral (AP to BL) fluxes of L-[1-14C]glucose (A) or 2-deoxy-d-[1-14C]glucose (B) were measured in intact pig tracheal epithelia over 1 hour. Data shown as mean ± s.e.m. n = 9 samples per group. (***: p<0.0001, ns: p ≥ 0.05). (C) Basolateral (BL) and apical (AP) uptake of 2-deoxy-d-[1-14C]glucose (2-DOG) in intact pig tracheal epithelia were measured over 1 hour. Data shown as mean ± s.e.m. n = 9 samples per group. (***: p<0.0001). (D) Correlation of transepithelial electrical conductance at baseline vs. transepithelial conductance of glucose in intact pig tracheal epithelia. In the intact tracheal epithelia of pigs, bidirectional fluxes of L-glucose were higher than in cultured human epithelia and fluxes of 2-DOG occurred at similar rates in the presence of uptake from both the apical and basolateral membranes. These data indicate that glucose supplied to intact airway epithelia can reach the ASL through the paracellular but not the transcellular pathway. Moreover, the result suggests that intracellular phosphorylation of glucose resulting in a transepithelial concentration gradient occurs at a higher rate in vivo compared to cultured cells. (TIFF) [file pone.0016166.s002.tif]

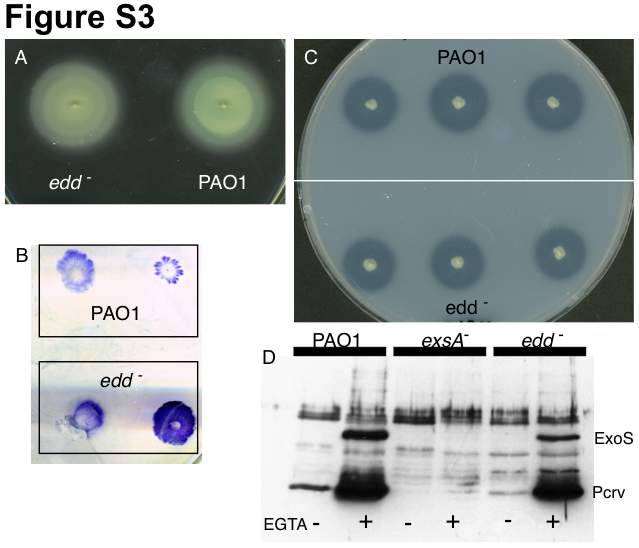

Supplement: Figure S3 — Swimming motility, twitching motility, protease activity and Type III secretion system protein expression are preserved in edd −. (A) A swimming motility assay comparing PAO1 and edd − was performed as described in [50]. Representative sample of n = 3 is shown. (B) A twitching motility assay comparing PAO1 and edd − was performed as described in [51], duplicate samples are shown. (C) Protease activity was characterized as described in [52], triplicate samples shown. (D) Expression of PcrV and ExoS, which are Type III secretion system proteins, was determined by using the method described in [53]. (TIFF) [file pone.0016166.s003.tif]

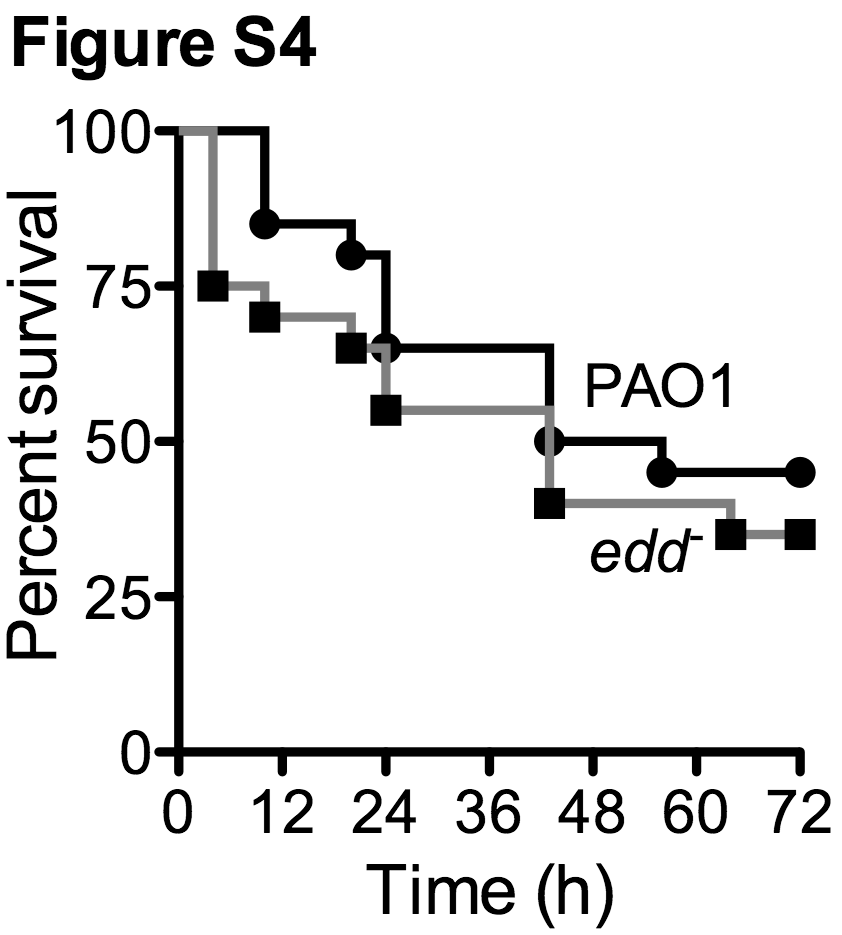

Supplement: Figure S4 — edd − lethality is preserved in a D. melanogaster infection model. Survival of D. melanogaster was monitored for 72 h after inoculation of the abdominal cavity with PAO1 or edd − as described in [54] (n = 20). (TIFF) [file pone.0016166.s004.tif]

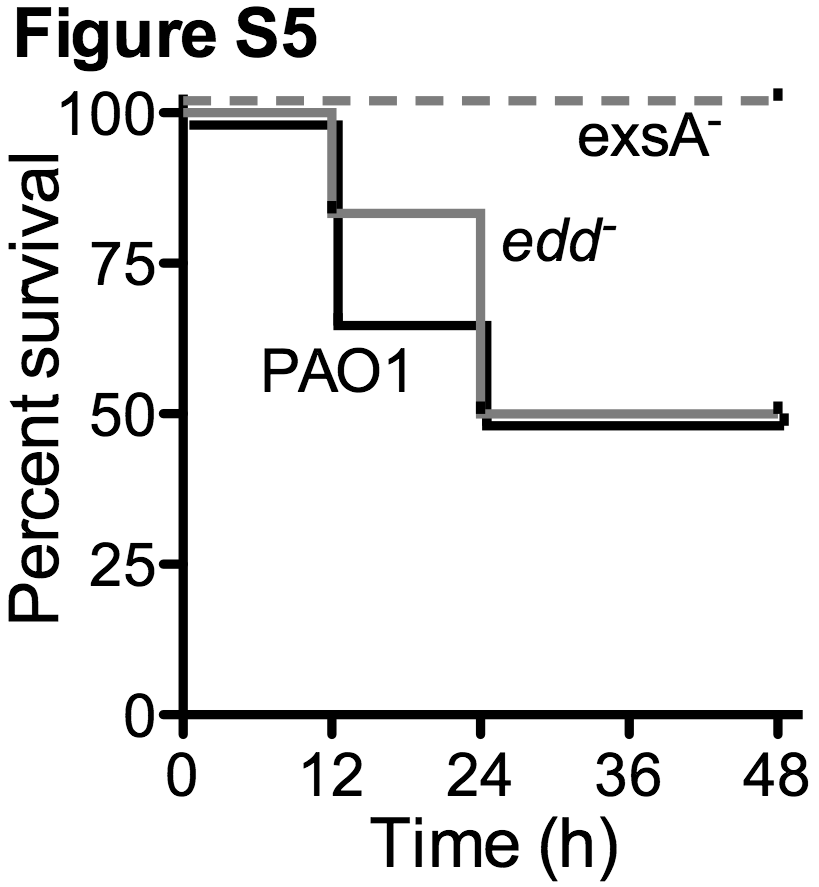

Supplement: Figure S5 — edd − lethality is preserved in a murine peritoneal infection model. All experiments were reviewed and approved by the Animal Care and Use Committee of the University of Iowa. Male 6 to 8 week old C57B/J6 mice obtained from Harlan Industries, Inc (Indianapolis, IN, USA) were used and were allowed access to food and water ad libitum. Mice were anesthetized with isoflurane and injected intraperitoneally with 3×10^7^ CFU of PAO1 or edd − in a volume of 100 μL H2O. exsA − strain of P aeruginosa was used as a negative control. Mice were carefully monitored and euthanized when end-point conditions were met (moribund, distressed, and unable to eat or drink). (TIFF) [file pone.0016166.s005.tif]
