# Supplementary material for: Glucose Depletion in the Airway Surface Liquid Is Essential for Sterility of the Airways
Source: PLoS One. 2011 Jan 20;6(1):e16166. doi: 10.1371/journal.pone.0016166 (PMC3029092; doi:10.1371/journal.pone.0016166)
Supplement: Text S1 — Supporting Information Methods. (DOC) [file pone.0016166.s006.doc]

**SUPPORTING METHODS**

**Primary cultures of human airway epithelia**

Cultures were used after at least 14 days of culture when they had developed morphologic and functional properties of airway epithelia and transepithelial resistance of 700-1200 Ω/cm2. Cultures were rinsed with phosphate buffered saline (GIBCO 14287-080, Grand Island, NY) 3 times and switched to antibiotic-free media for at least 7 days before conducting experiments.

**Transmission Electron Microscopy**

Primary cultures of human airway epithelia were gently immersed in 2% osmium tetroxide dissolved in FC-72 fluorocarbon (3M, Maplewood, MN). After two hours of fixation the samples were rinsed in pure FC-72, then immersed in 100% ethanol. Following two more 15 minute changes in ethanol the samples were transitioned to Eponate 12 (Ted Pella Inc. Redding, CA), embedded and then cured overnight at 70 °C. 80 nm thin sections were post stained with uranyl acetate and lead citrate and imaged in a JEOL 1230 transmission electron microscope (JEOL, Peabody, MA) Images were recorded using a 2k x 2k Gatan CCD camera (Gatan, Pleasanton, CA).

**Collection of airway surface liquid (ASL)**

M9 minimal medium (BD 248510, Franklin Lakes, NJ) was prepared according to manufacturer instructions and supplemented with 1mM MgSO4 and 100 μM CaCl2. For collection of ASL from 1 donor, 12 cultures were sequentially rinsed in the apical surface with a volume of 130 μL M9 medium to progressively concentrate ASL in the sample. Since a small volume of the collection media is unrecoverable after each rinse, the final volume of ASL sample is approximately 40 μL.

**Determination of ASL glucose concentration**

ASL was collected using M9 media with 300 μg/mL high molecular weight dextran conjugated to Alexa Fluor 488 (Molecular Probes D22910, Eugene, OR, USA). Samples were analyzed in a fluorescence spectrophotometer (Hitachi P-4500, Pleasanton, CA, USA) to compare initial fluorescence to final fluorescence. Fluorescence decreased 33.5% during collection, which shows that the sample is composed of 33.5% ASL. Glucose concentration (adjusted for dilution) in the sample was determined using the glucose protocol in the Amplex Red Glucose/Glucose Oxidase Assay Kit (Invitrogen A22189, Carlsbad, CA).

**Measurement of transepithelial electrical properties of intact human tracheal epithelia**

Tissues were mounted on tissue holding sliders (P2307), placed in a vertical Ussing chamber system (EasyMount P2300, Physiologic Instruments, San Diego, CA) and bathed on both sides by 5 mL of a solution containing (in mM): NaCl 135, K2HPO4 2.4, KH2PO4 0.6, CaCl2 1.2, MgCl2 1.2, and HEPES 5 and pH of solution adjusted to 7.2 with 10N NaOH. The solution was continuously gassed with hydrated air. Tissues were short-circuited using a multichannel voltage/current clamp (Physiologic Instruments Inc., model VCC MC8-4S, San Diego, CA, USA) and connected to a computerized data acquisition system (Acquire & Analyze 2.3.181). Apical amiloride 100 µM (Sigma-Aldrich, A7410, St. Louis, MO), GlyH 100 μM (Kind gift from Robert Bridges and Cystic Fibrosis Foundation Therapeutics), phlorizin 100μM (Sigma-Aldrich, P3449, St. Louis, MO), and glucose (1, 5 and 20 mM) were used as indicated.

**Primary antibodies**

Mouse anti-acetylated-Tubulin (Invitrogen 32-2700, Carlsbad, CA), mouse anti-E-Cadherin (Zymed 33-4000, South San Francisco, CA), rabbit anti-GLUT-1 (Abcam ab15309, Cambridge, MA), rabbit anti-GLUT-2 (Abcam ab54460, Cambridge, MA), rabbit anti-GLUT-3 (Abcam ab15311, Cambridge, MA), rabbit anti-GLUT-4 (Abcam ab654, Cambridge, MA), rabbit anti-GLUT-8 (Abcam ab35022, Cambridge, MA), rabbit anti-GLUT-10 (Abcam ab33245, Cambridge, MA), rabbit anti-SGLT-1 (Abcam ab14685, Cambridge, MA), rabbit anti-SGLT-2 (Abcam ab37296, Cambridge, MA). We did not investigate expression of GLUT-6 (pseudogene with no functional protein), GLUT-9 (urate transporter), GLUT-11 (skeletal and cardiac muscle fructose and glucose transporter), and GLUT-12 (Expressed predominantly in brain, skeletal and cardiac muscle, and kidney).

**Secondary antibodies**

Goat anti-rabbit Alexa fluor 488 (Invitrogen a11070, Carlsbad, CA), goat anti-rabbit Alexa fluor 568 (Invitrogen a11019, Carlsbad, CA).

**Bidirectional fluxes and uptake of glucose**

Primary cultures of HAE from 6 different donors were kept in glucose-free DMEM (Gibco 11966-025, Grand Island, NY) for 1 hour at 37 °C and 5% CO2 in a humidified incubator. 250 μL DMEM with 0.5 μCi 2-deoxy-d-[1-14C]glucose (55 mCi/mmol, GE Healthcare CFA562, Chalfont St. Giles, UK) and 5 mM 2-deoxyglucose (Sigma-Aldrich D8375, St. Louis, MO) or L-[1-14C]glucose (55 mCi/mmol, GE Healthcare CFA328, Chalfont St. Giles, UK) and L-glucose 5mM (Sigma-Aldrich G5500, St. Louis, MO) as indicated were added to the apical or basolateral compartment. 250 μL of glucose-free DMEM, adjusted for osmolality with xylitol (Sigma-Aldrich, X3375, St. Louis, MO) were added to the contralateral surface in each case. 15 μL samples were taken at baseline, 10, 30 and 60 min and added to scintillation cocktail (Research Products International Bio-safe II, Mount Prospect, IL) for radiotracer quantification in a liquid scintillation counter (Beckman Instruments LS6000SC, Fullerton, CA). Airway epithelia were rinsed 3 times with ice-cold PBS with phlorizin 200 μM and phloretin 100 μM (Sigma-Aldrich P7912, St. Louis, MO) and immersed in scintillation cocktail for 16 hours before quantification. Uptake of L-[1-14C] was used as background for correction of 2-deoxy-d-[1-14C]glucose uptake. Final volumes were measured for determination of water transport rate by epithelia and included in the analysis of transport rates.

**Measurement of transepithelial electrical properties in well-differentiated cultures of human airway epithelia.**

Human airway epithelia were mounted into Ussing chambers (Jim's Instruments, Iowa City, IA) and studied as previously described [S1]. Transepithelial voltage (referenced to the apical solution) was clamped at 0 mV using a 616C-2 dual voltage clamp (Bioengineering, University of Iowa, Iowa City, IA), and short-circuited current was recorded continuously. Transepithelial resistance was calculated from the change in current required to clamp the transepithelial voltage to +/- 5 mV by pulses delivered by a pulse generator built into the voltage clamp instrument. Epithelia were bathed in symmetrical solutions containing (in mM): NaCl 135, K2HPO4 2.4, KH2PO4 0.6, CaCl2 1.2, MgCl2 1.2, and HEPES 5 and pH of solution adjusted to 7.2 with 10N NaOH. The Ussing chamber solution was continuously gassed with hydrated 100% air. The following pharmaceuticals were used as indicated: amiloride (100 µM), bumetanide (100 µM) and glucose (0.5, 5 and 50 mM).

***P. aeruginosa* growth on cultures of human airway epithelia**

When indicated, cultures of human airway epithelia were preincubated with apical addition of 15 μL M9 medium with 1mM phloretin and 5mM glucose for 16 hours until clearance of apical fluid was evidenced. The required inoculum size of PAO1 was added to the apical side of cultures in 0.1 μL of M9 medium with either vehicle (ethanol), 20 mM glucose (Figure 4, C), or 1 mM phloretin (Figure 4, D) as indicated. Epithelia were kept in a humidified incubator at 37 °C with 5% CO2 for 24 h. The apical surface was rinsed with 100 μL PBS for bacterial counts. Under these conditions, counts at 24 hours were in all cases either > 7 log CFU or 0 CFU, not following a normal distribution, therefore each sample was categorized as either “sterile” or “infected” for analysis.

***P. aeruginosa* pulmonary challenge in mice**

Mice were allowed access to food and water *ad libitum*. Experiments were performed 2 hours after an intraperitoneal dose of glucose (2 mg/gr body weight) was given under isoflurane anesthesia. 10 μL of tail blood were obtained, and serum was used to determine glucose concentration with the Amplex Red Glucose Kit as described above. Mice were anesthesized with isoflurane and were inoculated intranasally [S2] with 50 μL containing 7.69 log CFU of PAO1 or *edd* - . After 6 hours, mice were euthanized and lungs were homogenized in a sterile tissue grinder (Kendall, 3505SA, Mansfield, MA) for bacterial counts in Difco Pseudomonas Isolation Agar (BD, 292710, Franklin Lakes, NJ).

**Bidirectional fluxes and uptake of glucose in intact tracheal pig epithelia.**

Epithelia dissected from tracheas of 3 to 4-week old pigs were mounted on tissue holding sliders (P2310, aperture area 0.2 cm2), placed in a vertical Ussing chamber system and analyzed under conditions as described in the transepithelial electrical properties section of Materials and Methods to obtain baseline open voltage and transepithelial resistance. After 10 minutes, the media was replaced with glucose-free DMEM for an incubation period of 10 minutes. Following incubation, media on either the apical or basolateral side was replaced with 2mL DMEM with 5mM L-glucose or 2-deoxyglucose as indicated and 4 μCi of 2-deoxy-d-[1-14C]glucose (Perkin-Elmer, NEC495050UC, Waltham, MA) or L-[1-14C]glucose (Perkin-Elmer, NEC478050UC, Waltham, MA). Media on the contralateral side was replaced with glucose-free DMEM containing xylitol to match osmolality of radiotracer media. 50 μL of media from both sides were obtained at baseline, 15, 30 and 60 minutes and radiotracer activity was quantified as described in the bidirectional fluxes and uptake of glucose section of Materials and Methods.

**SUPPORTING METHODS REFERENCES**

S1. Smith JJ, Karp PH, Welsh MJ (1994) Defective fluid transport by cystic fibrosis airway epithelia. J Clin Invest 93: 1307-1311.

S2. Seiler MP, Luner P, Moninger TO, Karp PH, Keshavjee S, et al. (2002) Thixotropic solutions enhance viral-mediated gene transfer to airway epithelia. Am J Respir Cell Mol Biol 27: 133-140.
